# Differential interactions of virulent and non-virulent *H. parasuis* strains with naïve or swine influenza virus pre-infected dendritic cells

**DOI:** 10.1186/1297-9716-43-80

**Published:** 2012-11-16

**Authors:** Tufária Mussá, Carolina Rodríguez-Cariño, Alejandro Sánchez-Chardi, Massimiliano Baratelli, Mar Costa-Hurtado, Lorenzo Fraile, Javier Domínguez, Virginia Aragon, María Montoya

**Affiliations:** 1 Centre de Recerca en Sanitat Animal (CReSA), UAB-IRTA, Campus de la Universitat Autònoma de Barcelona (UAB), 08193 Bellaterra, Barcelona, Spain; 2 Cátedra de Patología, Facultad de Ciencias Veterinarias, Universidad Central de Venezuela, Maracay, Venezuela; 3 Servei de Microscopia, Facultat de Biociències, Campus UAB, 08193 Bellaterra, Barcelona, Spain; 4 Universitat de Lleida, Lleida, Spain; 5 Dpto. de Biotecnología, INIA, Madrid, Spain; 6 Institut de Recerca i Tecnologia Agroalimentaria (IRTA), Barcelona, Spain

## Abstract

Pigs possess a microbiota in the upper respiratory tract that includes *Haemophilus parasuis*. Pigs are also considered the reservoir of influenza viruses and infection with this virus commonly results in increased impact of bacterial infections, including those by *H. parasuis*. However, the mechanisms involved in host innate responses towards *H. parasuis* and their implications in a co-infection with influenza virus are unknown. Therefore, the ability of a non-virulent *H. parasuis* serovar 3 (SW114) and a virulent serovar 5 (Nagasaki) strains to interact with porcine bone marrow dendritic cells (poBMDC) and their modulation in a co-infection with swine influenza virus (SwIV) H3N2 was examined. At 1 hour post infection (hpi), SW114 interaction with poBMDC was higher than that of Nagasaki, while at 8 hpi both strains showed similar levels of interaction. The co-infection with H3N2 SwIV and either SW114 or Nagasaki induced higher levels of IL-1β, TNF-α, IL-6, IL-12 and IL-10 compared to mock or H3N2 SwIV infection alone. Moreover, IL-12 and IFN-α secretion differentially increased in cells co-infected with H3N2 SwIV and Nagasaki. These results pave the way for understanding the differences in the interaction of non-virulent and virulent strains of *H. parasuis* with the swine immune system and their modulation in a viral co-infection.

## Introduction

*Haemophilus parasuis* is a non-motile, Gram-negative, small pleomorphic rod of the family *Pasteurellaceae*[1] and the causal agent of Glässer’s disease, which is characterized by fibrinous polyserositis and polyarthritis [2,3]. Glässer’s disease is common in all countries with commercial pig production and its economic cost is considered one of the highest in nursery pigs, in which the disease is more prevalent [4]. Fifteen serovars of *H. parasuis* have been defined, with serovars 1, 5, 10, 12, 13 and 14 classified as highly virulent; serovars 2, 4 and 15 as moderately virulent; and serovars 3, 6, 7, 8, 9 and 11 as non-virulent [5]. The pathogenesis of *H. parasuis* remains unclear, but disease production is influenced by stress, the presence of virulent strains of *H. parasuis* and other pathogens in the farm and immune status of the animals [6]. Differences between virulent and non-virulent strains have been determined at the genetic level [7], in functional assays of phagocytosis [8], serum resistance [9], adhesion and invasion of endothelial cells [10,11] and, more recently, in adhesion and invasion to epithelial cells [12]. However, *H. parasuis* interaction with porcine DC has not been addressed before, neither has its possible immunomodulation in the presence of other respiratory pathogens such as swine influenza virus (SwIV).

Influenza viruses are enveloped, single stranded RNA (ssRNA) viruses in the *Orthomyxoviridae* family. Pigs play a crucial role in the interspecies transmission of influenza viruses [13-15]. The pathogenicity of influenza virus lies in its ability to elude host anti-viral immune responses. In pigs, primary SwIV infection induced cytokines like IFN-α, IL-6, IL-1, TNF-α, IFN-γ and IL-12, which have been associated with acute influenza virus infection in pigs [16].

An efficient immune response against a particular pathogen depends on the efficient recognition of the pathogen/antigen, and dendritic cells (DC) play an essential role in priming this effective immune response. They constitute the bridge between the innate and adaptive immune response [17]. According to their functionality and phenotype, DC can be classified as conventional DC (cDC) known as professional presenting cells or plasmacytoid DC (pDC), which naturally produce high levels of type-I interferon [18]. In swine, both cDC and pDC have important antigen-presenting functions and they complement each other by having distinct regulation of major histocompatibility complex class I (MHC-I, SLA-I in swine) and class II (MHC-II, SLA-II in swine) depending on antigen presentation and different profile of secreted cytokines [19]. Interestingly, conventional DC are amongst the first cells encountered by most viruses, simply due to their availability at every possible entry site of the body [20]. Respiratory disease in pigs is common in modern pig production worldwide and is often referred to as porcine respiratory disease complex. This disease complex results from infection with various combinations of primary and secondary respiratory pathogens, including *H. parasuis* and SwIV [21,22]. Our main goal was to assess whether *H. parasuis* virulence implied a differential interaction with swine DC and whether this interaction would be altered in the presence of another respiratory pathogen involved in the porcine respiratory disease complex, such as SwIV, considering all this previous knowledge and the availability of DC beneath the epithelium of respiratory organs. Here, we report different patterns of interaction and activation of swine DC after encountering virulent and non-virulent *H. parasuis* strains which was in turn altered in the presence of swine influenza virus. These results will help understanding how bacteria-viral infection influences the outcome of the elicited immune response.

## Material and methods

### Porcine bone marrow dendritic cells (poBMDC)

Bone marrow hematopoietic cells were obtained from femurs of eight-week old healthy Large white X Landrace pigs, negative for porcine reproductive and respiratory syndrome virus (PRRSV) and type-2 porcine circovirus (PCV2) by RT-PCR as previously described by Olvera et al. and Sibila et al. [23,24]. These animals were also negative by enzyme linked-immunosorbent assay (ELISA) for influenza virus and *Actinobacillus pleuropneumoniae* (HIPRA, Amer, Spain), for Mycoplasma (OXOID, Cambridge, UK), for Parvovirus, Adenovirus and Aujeszky’s disease virus (INGENASA, Madrid, Spain), and Salmonella (SVANOVA Biotech AB, Uppsala, Sweden). Bone marrow dendritic cells (BMDC) were generated using an eight day protocol as previously described by Mussá et al. and Carrasco et al. [25,26]. Briefly, bone marrow haematopoietic cells (BMHC) were resuspended in RPMI-1640 (Lonza, Walkesville, USA) culture medium containing 2 mM of L-glutamine (Invitrogen®, Barcelona, Spain), 100 U/mL of Polymixin B (Sigma-Aldrich, Madrid, Spain) 10% of fetal calf serum (FCS) Euroclone, Sziano, Italy) and 100 U/mL of penicillin with 100 μg/mL of streptomycin (Invitrogen®, Barcelona, Spain). Every 3 days new medium containing 100 ng/mL of rpGM-CSF (R&D Systems, Madrid, Spain) was added. At day 8 of generation floating and semi-adherent cells were harvested, washed in RPMI with L-glutamine only and used in the proposed experiments. Animal care and procedures were in accordance with the guidelines of the Good Laboratory Practices (GLP) under the supervision of the Ethical and Animal Welfare Committee of the Universitat Autònoma de Barcelona (number of approval: 1189) and under the supervision of the Ethical and Animal Welfare Committee of the Government of Catalonia (number of approval: 5796).

#### Haemophilus parasuis

Two reference strains of *H. parasuis*, SW114 and Nagasaki, belonging to the nasal and systemic clade respectively on the multilocus sequence typing (MLST) were used to inoculate poBMDC. SW114 is the serovar 3 reference strain (non-virulent); Nagasaki is the serovar 5 reference strain (highly virulent) [5,27]. For DC inoculation, bacteria were grown overnight as described by Olvera et al. [8]. Briefly, strains were cultured on chocolate agar plates (BioMérieux, Madrid, Spain) overnight at 37°C and 5% CO_2_. The following day, colonies were collected and resuspended in PBS at the appropriate concentration. Bacterial counts in the inocula were confirmed by plating serial dilutions on chocolate agar plates.

### Influenza virus

Porcine A/swine/Spain/SF32071/2007(H3N2) SwIV strain was isolated from a natural outbreak in a conventional farm in 2007, in Spain. Eight sequences of this virus, corresponding to HA, NP, PA, PB2, NA, PB1/ PB1-F2, NS1/NS2 and M1/M2 genes were submitted to GenBank (accession numbers: HE774666, HE774667, HE774668, HE774669, HE774670, HE774671, HE774672 and HE774673). Virus isolation and SwIV-cell infection were performed as previously described by Mussá et al. [26] except for the fact that the post infection medium did not contain any antibiotics.

### DC infection or stimulation with Poly:IC

**Figure 1:**
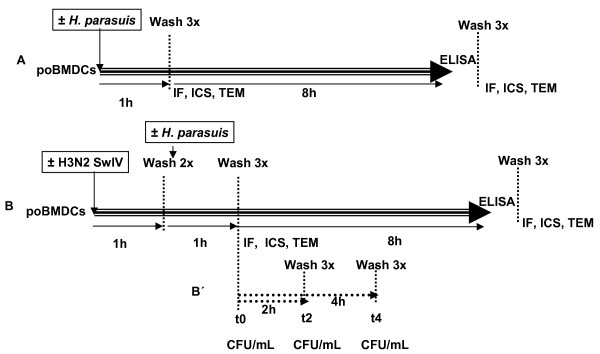
**Experimental design.** (**a**) *H. parasuis* single infection. At day 8 of differentiation, poBMDC were infected with SW114 or Nagasaki. Infected cells were collected at 1h and 8 hpi for IF, ICS or fixed for TEM studies. At 8 hpi, supernatants were collected for cytokine screening by ELISA. (**b**) Co-infection of poBMDC with H3N2 SwIV and *H. parasuis*. Porcine BMDC were infected with H3N2 SwIV for 1h followed by *H. parasuis* for another 1h. After that cells were left for a further 8h to obtain supernatants for ELISA. Cells for IF, ICS and TEM were collected at 1h and 8 hpi. (c) Intracellular survival assay. After 1h of co-infection (time zero), the cells were washed and incubated for a further 2 or 4h with medium containing gentamicin and penicillin G. At t0, t2 and t4, cells were disrupted with water to quantify the CFU/mL. IF- immunofluorecence, ICS- intracellular staining, TEM- transmission electron microscopy.

After 8 days of generation, poBMDC were harvested and washed with RPMI with neither antibiotic nor serum, counted and plated on 24 well plates (Figure 1a and 1b). Then, 10^7^ CFU (MOI 10) of SW114 or Nagasaki were added to the wells. After 1h of incubation at 37°C with the bacteria, cells were washed thrice with RPMI by centrifugation at 450 × *g* for 5 min. After the third wash the supernatants were discarded, 500 μL of RPMI containing L-glutamine and 10% FCS were added and plates were incubated for further different times at 37°C 5% CO_2_. While at 1 hpi with bacteria, cells were washed, harvested and stained, at 8 hpi and the supernatants were frozen at −20°C for cytokine detection by ELISA (see below) and the cells were harvested for staining. When required, infection with H3N2 SwIV was performed as follows: one hundred microliters with 10^4^ TCID_50_ of swine influenza H3N2 virus were added and left to adhere for 1h at 37°C 5% CO_2_. After that, cells were washed once with RPMI and incubated with medium only or with *H. parasuis* as stated previously. To analyse the effect of toll like receptor 3 (TLR3) in *H. parasuis* infection, the cells were stimulated with 50 μg/mL of Polyinosinic:Polycytidilic acid salt (Poly: IC) (Sigma-Aldrich, Madrid, Spain) for 4h before infection with *H. parasuis* and then left for a further 5h.

### Staining of *H. parasuis*

*H. parasuis* was assessed by flow cytometry or by immunofluorescence at 1h and at 8 hpi (Figure 1a and 1b). For flow cytometry, poBMDC were fixed with 4% paraformaldehyde (EMS, Hatfield, PA, USA) for 10 min at room temperature (RT). After washing (centrifugation 450 × *g* for 5 min at 4°C), cells were permeabilized with 0.1% Triton-X100 in PBS for 15 min at 37°C. Then, 1h incubation at 4°C for each antibody was used. A polyclonal rabbit anti-SW114 or anti-Nagasaki serum (10 μL serum/490 μL PBS 2% FCS) was used as primary antibody. An FITC-conjugated monoclonal anti-rabbit (Clone RG-16, Sigma-Aldrich, Madrid, Spain) was used as a secondary antibody at 1:100 dilution. For immunofluorescence, the cells were infected on a circular glass cover slip (VWR International, Barcelona, Spain) inserted in 24 well plates (Nunc®, Kamstrupvej, Denmark). After infection, supernatants were collected for ELISA. Then, the cells were fixed with ethanol (Panreac, Barcelona, Spain) for 10 min at 4°C, dehydrated with acetone and then permeabilized with 0.1% Triton X-100 for 15 min at 37°C. Then, the cells were washed with PBS with 0.1% BSA and 100 μL of polyclonal rabbit anti-SW114 or anti-Nagasaki serum diluted as before were added and the slides were incubated for 1h at 4°C. After three washes, a secondary antibody anti-rabbit Immunoglobulin-FITC diluted 1:50 was added and incubated for 1h at 4°C. Phalloidin, Tetramethylrhodamine B isothiocyanate (TRITC) (Sigma-Aldrich, Madrid, Spain) at 5 μg/mL was used to stain actin filaments situated beneath the cell membrane and finally after several washes, nuclei were counterstained with DAPI. Cover slips were dried and mounted using 1 drop of Fluoprep (BioMérieux, Madrid, Spain). To detect autofluorescence, mock or infected-poBMDC were stained as controls with the primary and/or secondary antibody. Treated cells were viewed on a Nikon eclipse 90i epifluorescence microscope equipped with a DXM 1200F camera (Nikon Corporate, Japan). To assess the association of bacteria with poBMDC, image stacks were captured using a Leica TCS SP2 confocal microscope, with an objective of 63×. Z stack images were acquired at intervals of 0.3 μm. Images were processed by using the ImageJ v1.42k software [28].

### Transmission electron microscopy (TEM)

Mock and infected poBMDC were fixed with 2% (w/v) paraformaldehyde (EMS, Hatfield, PA, USA) and 2.5% (v/v) glutaraldehyde (EM grade, Merck, Darmstadt, Germany) in 0.1 M phosphate buffer (PB, Sigma-Aldrich, Steinheim, Germany), pH 7.4 and processed following conventional procedures as previously described in detail by Rodriguez-Cariño et al., [29]. Briefly, the samples were post-fixed with osmium, dehydrated in acetone, embedded in Epon, polymerized at 60°C and cut with an ultramicrotome. Finally, ultrathin sections placed in copper grids were contrasted with conventional uranyl acetate and Reynolds lead citrate solutions and observed using a Jeol 1400 (Jeol LTD, Tokyo, Japan) transmission electron microscope equipped with a CCD GATAN ES1000W Erlangshen camera.

### Intracellular survival assay

Cells were infected as described above. After 1 h of *H. parasuis* infection (time zero) (Figure 1b and 1c), RPMI containing 2mM of L-glutamine and 10% of FCS, penicillin G (5 μg/mL) and gentamicin (100 μg/mL) (both from Sigma-Aldrich, Madrid, Spain) was added and the poBMDC were left at 37°C and 5% CO_2_ for a further two or four hours. At these times, after three washes with PBS, poBMDC were disrupted using sterile water, and serial 10 fold dilutions of lysate were plated on chocolate agar and incubated for 48 h. At the same times, 100 μL of cell culture supernatants were plated on chocolate agar (Biomérieux, Madrid, Spain) to check if *H. parasuis* was efficiently killed by the antibiotics.

### Activation markers

Fifty microliters of poBMDC (5 × 10^5^ cells) were plated in 96 well U-bottom plates (Nunc® Kamstrupvej, Denmark) and washed with PBS 2% FCS. Then, 100 μL hybridoma supernatants containing anti-SLA-I (4B7/8) or anti-SLA-II (1F12), and the human CTLA4 murine immunoglobulin fusion protein (CTLA4-muIg; Ancell, Bayport, Minnesota, USA) were used as primary antibodies. The secondary antibody was R-phycoerythrin anti-mouse IgG (Jackson ImmunoResearch, Suffolk, UK). Stained cells were acquired using a Coulter® EPICS XL-MCL cytometer and analysed with the EXPO 32 ADC v.1.2 program. The mean fluorescence intensities (MFI) of each sample were analysed.

### Enzyme linked-immunosorbent assay (ELISA)

The supernatants of 8 h infected-poBMDC were thawed only once for cytokine detection by ELISA. All ELISA were read with KC Junior Program (BioTek, Potton, UK) using the filter PowerWave XS reader. For IL-6, IL-1β, IL-10 and TNF-α, the Duo Set Developed ELISA system from R&D System® was used following the manufacturer’s instructions while for IFN-α an in house ELISA using antibodies purchased from PBL Interferon Source was used according to Kekarainen et al. [30]. To detect IL-8 and IL-18, swine IL-8 (CXCL8) VetSet™ ELISA development kit (Kingfisher Biotek, Saint Paul, MN, USA) and pig IL-18 Module Set BMS672MST (Bender Med Systems, Vienna, Austria) were used following the manufacturer’s instructions. Finally IL-12 secretion was analysed using antibodies anti IL-12/IL-23p40 (R&D Systems, Madrid, Spain) according to the manufacturer’s instructions with the following amounts of antibodies: 2 μg/mL of anti IL-12/IL23 monoclonal antibody were used to coat a 96 well plate (Costar, NY, USA) overnight at RT. After washing, serial dilutions of the recombinant porcine IL-12 starting from 10 000 pg/mL were added. Then 125 ng/mL of biotinylated anti-porcine IL-12/IL-23 p40 antibody was used. Finally, 0.05 μg/mL of peroxidase-conjugated streptavidin (Jackson ImmunoResearch, Suffolk, UK) was added. The reaction was revealed using 3,3’,5,5’ tetramethylbenzidine (TMB) (Sigma-Aldrich, Madrid, Spain) and stopped using H_2_SO_4_ (0.5M). The limits of detection were 3.9 U/mL and 78.1 pg/mL for IFN-α and IL-12 respectively.

### Statistical analysis

All statistical analyses were performed using SPSS 15.0 software (SPSS Inc., Chicago, IL, USA). For statistical comparisons, the pig (as source of cells) was used as the experimental unit. The significance level (α) was set at 0.05. A non-parametric test (Mann–Whitney) was chosen to compare the different values obtained for all the immunological parameters between groups at all sampling times.

## Results

### Differential interaction of *H. parasuis* Nagasaki and SW114 individually or in SwIV co-infection with swine DC

**Figure 2:**
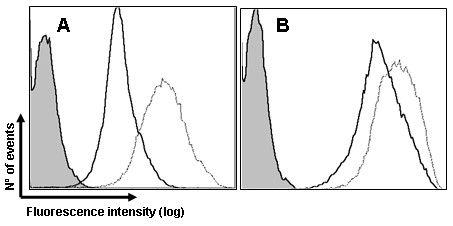
**Staining of *H. parasuis *SW114 or Nagasaki at 1 h (a) and 8h (b) after infection.** Porcine BMDC were infected and stained using anti-SW14 or anti-Nagasaki rabbit serum for 1 h at 4°C, and then with the anti-rabbit IgG-FITC antibody. Mock (grey histograms), SW114 (dotted line), Nagasaki (continuous line). Representative of independent experiments using poBMDC derived from four animals.

Measuring *H. parasuis* interaction with swine DC may be a suitable indicator of virulence since DC ability to capture antigens is important to elicit a specific immune response. At 1 hpi poBMDC infected with SW114 or Nagasaki showed higher fluorescence intensity compared to mock cells (Figure 2a), however, at 8 hpi it seems that both strains interacted equally with poBMDC compared to the mock cells (Figure 2b).

**Figure 3:**
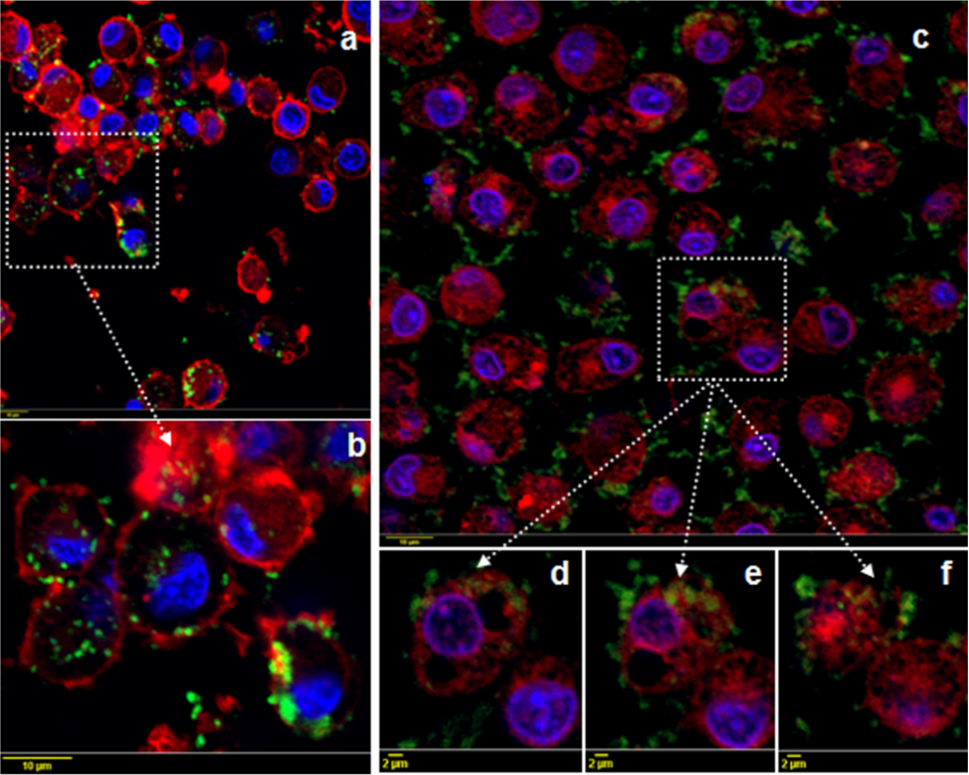
**Confocal images of poBMDC infected with SW114 for 1h (a–b) and 8h (c–f).** Porcine BMDC were stained with polyclonal anti-SW14 rabbit serum and anti-rabbit IgG-FITC antibody. Cytoplasm was stained with rhodamine-phalloidin and nuclei with DAPI. Green (SW114), red (cytoplasm), blue (nuclei). Bars a,b and c = 10 μm, while bars d-f = 2 μm. b and d-f are representative results of Z stack sections.

**Figure 4:**
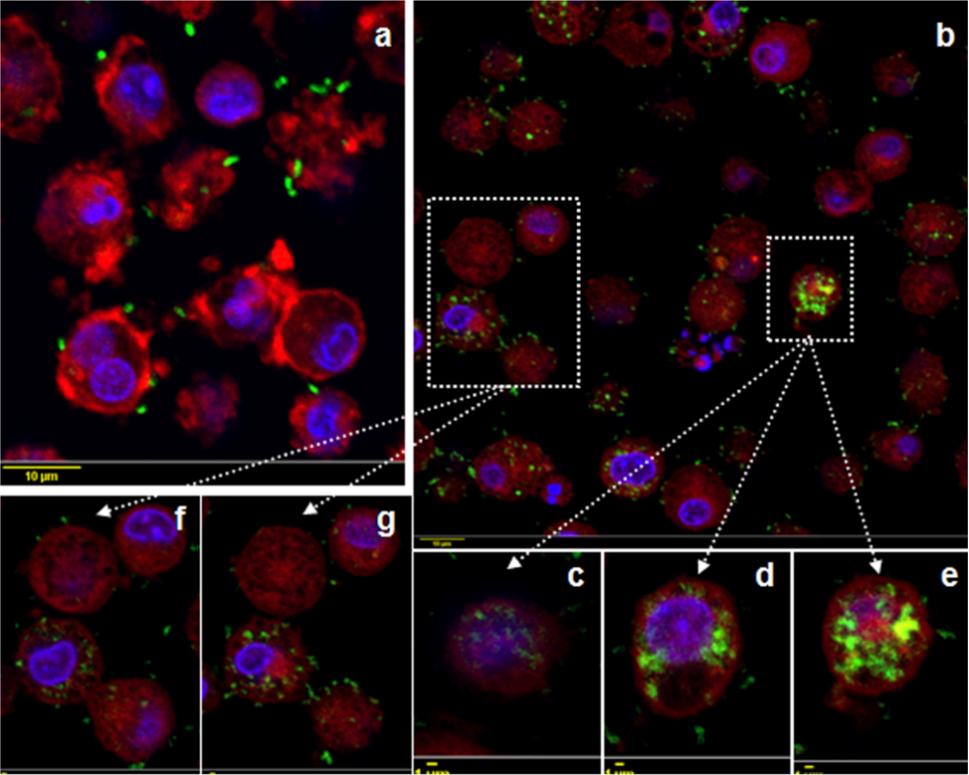
**Confocal images of poBMDC infected with Nagasaki for 1h (a) and 8h (b–g).** Porcine BMDC were stained with polyclonal anti-Nagasaki rabbit serum and anti-rabbit IgG-FITC antibody. Cytoplasm was stained with rhodamine-phalloidin and nuclei with DAPI. Green (Nagasaki), red (cytoplasm), blue (nuclei). Bars a and b = 10 μm, while bars c-g = 1 μm. c-g are representative results of Z stack sections.

The ability of poBMDC to interact with and internalise *H. parasuis* was confirmed by confocal and electron microscopy. Confocal microscopy was performed using serum against each particular strain. One hour after infection, the SW114 strain was observed attached and inside poBMDC (Figure 3a and 3b) and at 8 hpi bacterial numbers increased inside the cell (Figure 3c and 3d–f). Conversely, the Nagasaki strain was mostly attached to the cells 1 hpi, apparently in fewer numbers (Figure 4a). At 8 hpi, Nagasaki was observed not only attached but also inside the DC (Figure 4b and 4c–g).

**Figure 5:**
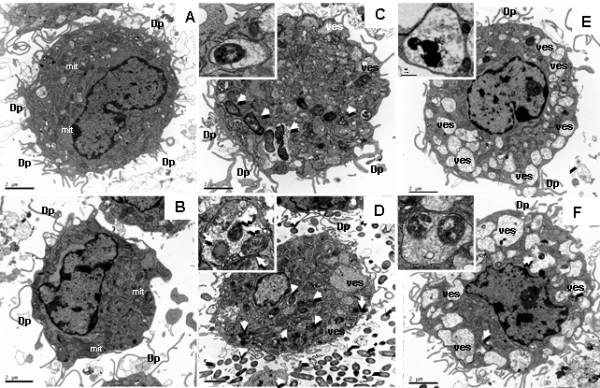
**TEM of mock poBMDC or *H. parasuis *infected-poBMDC at 1hpi (a,b,c) and at 8 hpi (d,e,f). a** and **d**: Mock poBMDC with normal morphology of DC, dendritic processes (Dp) as well as some organelles such as mitochondria (mit). **b** and **e**: SW114-infected- poBMDC, b: poBMDC with several vesicles in the cytoplasm (ves) some of them containing bacteria (white-arrows indicate some of the bacteria). Bacteria were inside phagolysosomes and some had a degenerated aspect (more electron dense); in the upper-left box, details of bacteria can be observed bounded to phagosome membrane. e: 8 h later, more SW114 bacteria were appreciated inside poBMDC (white-arrows and close-up). **c** and **f**: Nagasaki- infected- DCs. **c**: 1 hpi, there were several vesicles (ves) in the cytoplasm, of different sizes and, some contained bacteria (white-arrow) as can be noted in the upper-left box. **f**: 8h later, the number and size of vesicles (ves) increased and also the Nagasaki bacteria were found inside vesicles (white-arrow) within the cell (close-up). Bar = 2 μm.

The interaction of the two strains with poBMDC was also analysed at the ultrastructural level by electron microscopy. At 1 or 8 hpi, mock poBMDC showed normal morphology (Figure 5a and 5d). In agreement with the fluorescence microscopy results, differential interaction with poBMDC was observed after SW114 or Nagasaki infection: at 1 hpi, higher numbers of SW114 bacteria were found inside phagolysosome-like structures as compared with Nagasaki infected cells (Figure 5b). At 8 hpi, more than one SW114 bacteria were found inside phagolysosome-like structures (Figure 5e). Infection of poBMDC with Nagasaki induced more apparent subcellular changes. Although, few bacteria were observed inside the poBMDC after the first hpi, an important number of vesicles were observed (Figure 5c). These changes were more evident at 8 hpi. At this time, larger vesicles containing more than one Nagasaki bacteria were observed (Figure 5f).

**Figure 6:**
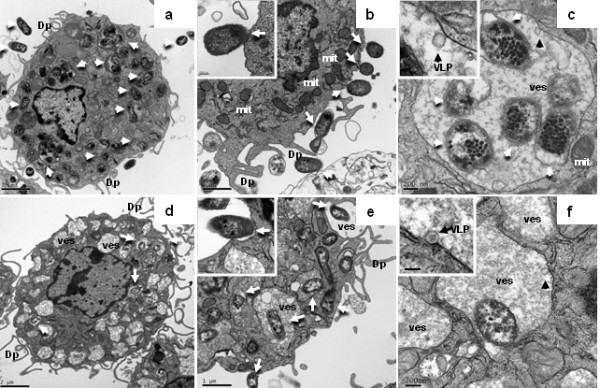
**TEM of poBMDC co-infected with H3N2 SwIV and *H. parasuis *at 8 hpi.** a, b and c: H3N2 SwIV plus SW114 strain. a: Several bacteria were observed inside the poBMDC (white-arrow) occupying phagolysosomes, and few vesicles, Bar = 2 μm. b: Many SW114 bacteria were observed attached to the cellular membrane (white-arrow), as can be observed in the close-up (left-up box), Bar = 1 μm. c: In some phagolysosomes which contained bacteria (white-arrow) also virus-like particles (VLP) were observed, (black-arrow) 100 nm in diameter, Bar = 0.5 μm. d, e and f: H3N2 SwIV plus Nagasaki strain. d: Few Nagasaki bacteria (white-arrow) were seen in poBMDC. However, many vesicles of different sizes which occupied large sub-cellular space were observed; less organelles were seen, Bar = 2 μm. e: Some poBMDC showed a large number of bacteria (white-arrow) inside and outside. Nagasaki was also, to a lesser extent, attached to the cellular membrane, Bar = 1 μm. f: This figure shows a phagolysosome-like structure with Nagasaki strain and VLP (black-arrow) inside, in the upper-left box VLP can be observed. Bar = 0.2 μm.

To get insight into the changes in the interaction of DC with *H. parasuis* as a consequence of a previous infection by SwIV, in vitro co-infection experiments were performed. The behaviour of poBMDC to *H. parasuis* infection when a previous infection with H3N2 SwIV took place was analysed by flow cytometry (Additional file 1: Figure S1), fluorescence microscopy (Additional file 2: Figure S2) and TEM (Figure 6). As previously observed, both *H. parasuis* strains showed differences in their interaction with poBMDC in the same range, even when SwIV pre-infection was present: H3N2 SwIV infected DC exhibited a higher level of interaction with SW114 than the Nagasaki co-infected cells at 1 hpi (Additional file 1: Figure S1) and at 8 hpi (Figure 6a–b and 6d–e). In these experiments, co-infected poBMDC presented SwIV virus-like particles inside vesicles. On some occasions SW114 or Nagasaki were in the same phagolysosome-like structures as these SwIV virus-like particles (Figure 6c and 6f). At 8 hpi, SW114 co-infected cells showed many bacteria inside phagolysosome-like structures which might define different levels of degradation (Additional file 3: Figure S3d–g). Moreover, the organelles of Nagasaki co-infected cells showed important changes such as dilation of the Golgi complex (Additional file 4: Figure S4f, S4i) and degraded bacteria inside phagolysosome-like structures (Additional file 4: Figure S4h).

### Intracellular survival of *H. parasuis* in poBMDC

**Figure 7:**
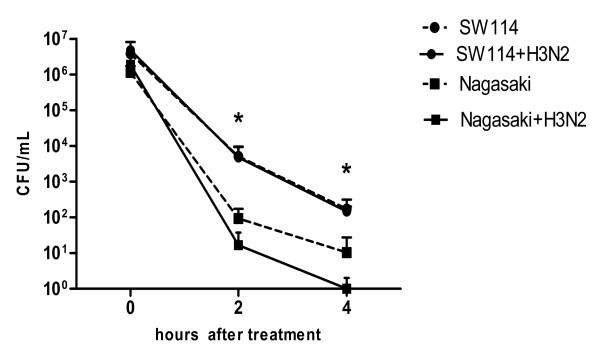
**Intracellular survival assay of *H. parasuis.*** Porcine BMDC were infected or co-infected with H3N2 SwIV and SW114 or Nagasaki. After 1 h (time zero; adherent and internalised bacteria), cells were incubated with gentamicin and penicillin G for 2 or 4 h. After each time, cells were disrupted and 100 μL plated on chocolate agar for 48 h. Two replicas of each condition were processed in each experiment. Bars indicate mean ± SEM of independent experiments using poBMDC derived from three animals. (*) indicate significant differences between groups at 2 h (*p* < 0.03) and 4 h (*p* < 0.04).

The above-mentioned results suggest that bacterial numbers differed for both *H. parasuis* strains in DC. To analyze the intracellular fate of bacteria once internalized, we quantified bacterial intracellular survival over time. Precisely, after 60 min incubation of *H. parasuis* with DC to get optimal internalisation, antibiotics were added and the treatment was lengthened for different times up to 4 h (Figure 1c). Before antibiotic addition, mean values of SW114 viable bacteria were 3.8 × 10^6^ CFU/mL while for Nagasaki they were 1.1 × 10^6^ CFU/mL. As shown in Figure 7, once internalised, the number of viable bacteria significantly decreased at time 2 h and 4 h (0.03 >*p* < 0.04) when compared to time zero in all tested groups (Figure 7). Differences between SW114 and Nagasaki in their interaction with poBMDC were noteworthy, with the number of SW114 being significantly higher than the number of Nagasaki.

### Activation profile of *H. parasuis*-SwIV-infected DC

**Figure 8:**
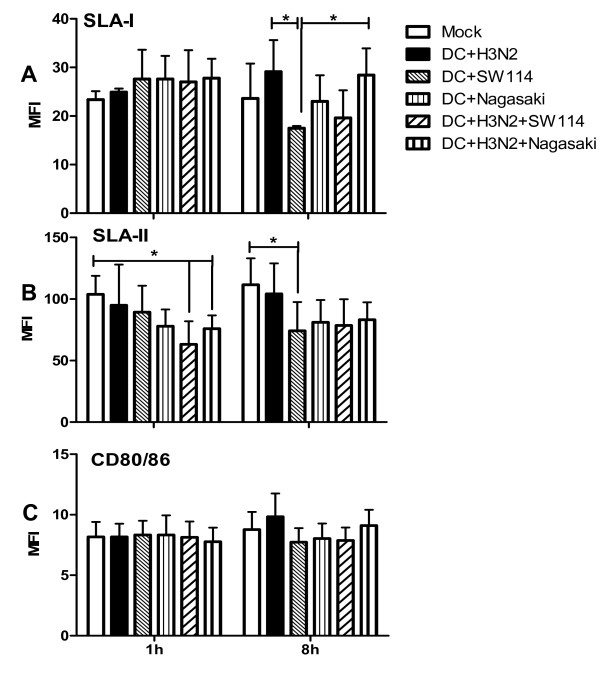
**Expression of (a) SLA-I, (b) SLA-II and (c) CD80/86 at 1 h and 8 hpi.** After each analysed time, mock and infected-poBMDC were stained using mAb anti-SLA-I, anti-SLA-II or human CTLA4 murine immunoglobulin fusion protein (CTLA4-mIg) for CD80/86. The mean fluorescence of each marker was analysed. Asterisk (*) indicate significant difference between the compared groups (*) *p* < 0.05. Representative results of independent experiments using poBMDC derived from three animals.

One of the hallmarks of DC is the triggering of an effective immune response by capture, processing and presentation of antigens to T cells. The antigen presenting process needs a complete signalling, where MHC presenting molecules (signal one), activation or costimulatory (signal two) and soluble mediators such as cytokines (signal three) participate. Therefore, to evaluate the level of expression of activation markers, poBMDC were stained for SLA-I, SLA-II and CD80/86 after infection with SW114 or Nagasaki. After 1 h of infection with *H. parasuis*, no significant differences in SLA-I, SLA-II or CD80/86 expression were found with any *H. parasuis* strains (Figure 8). Statistically significant decreases (*p* < 0.05) were found at 8 hpi in SLA-I and SLA-II of SW114-infected poBMDC compared to SwIV infected cells or mock respectively (Figure 8a and 8b). No significant differences were observed in CD80/86 expression (Figure 8c).

Expression levels of activation markers were also analysed after co-infection. One hour after H3N2 SwIV single infection or H3N2 SwIV-SW114 and H3N2 SwIV-Nagasaki co-infections, no significant differences (*p* > 0.05) were observed in SLA-I and CD80/86 expression. However, at this time, significant decreases (*p* < 0.05) of SLA-II were observed in H3N2 SwIV plus SW114 or Nagasaki co-infected cells (Figure 8b). At 8 hpi, only H3N2 SwIV single infection or co-infection with Nagasaki induced a statistically significant increase of SLA-I when compared to SW114 single infection (Figure 8a).

### Cytokine pattern of *H. parasuis* infected DC: effects of SwIV co-infection

**Figure 9:**
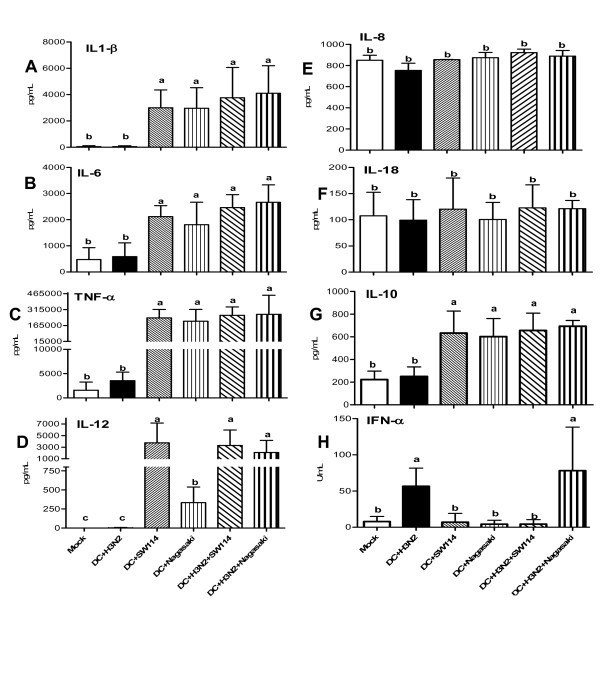
**Cytokine secretion by poBMDC at 8 hpi.** poBMDC were infected or co-infected for 8 h with H3N2 SwIV and SW114 or Nagasaki. Supernatants of mock, single or co-infection were analysed for cytokine secretion using ELISA. Bars are mean ± SEM of three independent experiments using poBMDC derived from three animals. The presence of significant differences (*p* < 0.05) between groups are represented by (a, b or c).

Once the profile of poBMDC activation after *H. parasuis* infection was characterised, we analysed the cytokine profile to determine the type of immune response that these pathogens may induce in poBMDC. Consequently, IL-1β, TNF-α, IL-6, IL-10, IL-8, IL-12, IL-18 and IFN-α were analysed at 8 hpi. Statistically significant differences (*p* < 0.05) were found in the secretion of IL-1β, IL-6, TNF-α and IL-10 between SW114 and Nagasaki-infected poBMDC compared to mock cells. IL-12 was secreted in higher levels by SW114-infected cells compared to Nagasaki-infected cells and to mock cells (Figure 9). No significant differences were found in IL-8, IL-18 or IFN-α secretion from SW114 or Nagasaki infected cells compared to the mock controls (Figure 9e, 9f and 9h).

Co-infection of poBMDC with H3N2 SwIV and SW114 or Nagasaki resulted in similar levels in most of the tested cytokines. However, a significant increase in the secretion of IL-12 compared to mock control cells was observed at 8 hpi in Nagasaki co-infected cells (*p* < 0.05) (Figure 9d). IL-12 production in Nagasaki infected-poBMDC was modulated by a previous H3N2 SwIV virus infection, while induction of IL-12 by SW114 was not affected. H3N2 SwIV induced higher levels of IFN-α compared to mock control cells. Surprisingly, a significant decrease of this cytokine was observed in H3N2 SwIV-SW114 co-infected cells compared to H3N2 SwIV infected cells whereas the Nagasaki strain significantly upregulated IFN-α secretion in co-infected cells compared to Nagasaki single infected cells (Figure 9h).

### Poly:IC immunomodulation on *H. parasuis* infected DC

**Figure 10:**
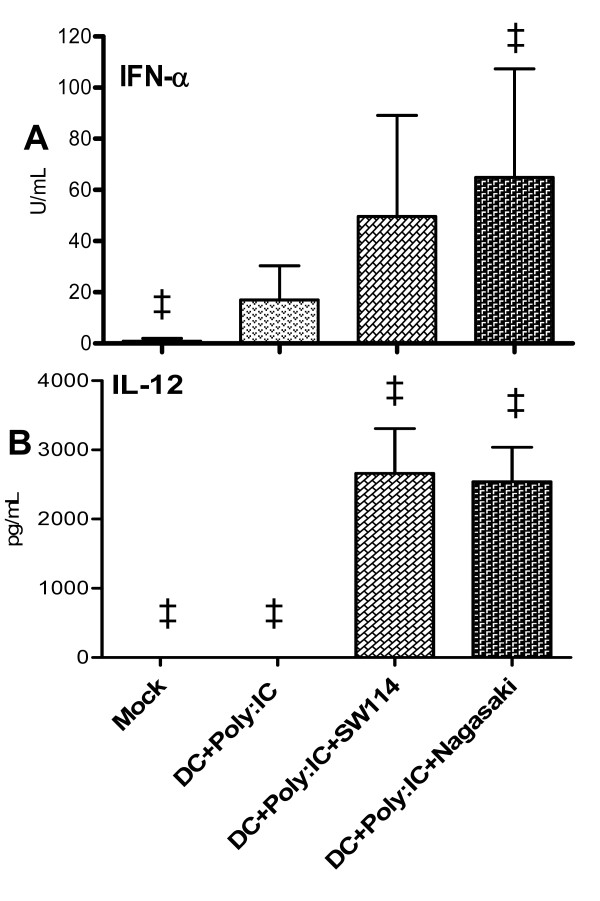
**IFN-α (a) and IL-12 (b) secretion by poBMDC stimulated with Poly:IC before *H. parasuis *infection.** Porcine BMDC were stimulated with Poly:IC for 4 h and then infected with SW114 or Nagasaki for a further 5 h. Supernatants of mock and stimulated, samples were analysed for IFN-α and IL-12 secretion using ELISA. Statistical tendencies were observed (‡) with *p* = 0.09 in (a) and *p* = 0.1 in (b). Bars are means ± SEM of two independent experiments using poBMDC derived from two animals.

The differences in IL-12 and IFN-α secretion by cells infected with SW114 or Nagasaki strains and the modulation of both cytokines in SwIV-*H. parasuis* co-infected DC led us to investigate whether other stimuli, like Poly I:C as a surrogate of an RNA viral infection, could alter this cytokine pattern in the same way. Hence, DC were pre-treated for 4h. with Poly:IC, before infection with *H. parasuis*. Lower levels of IL-12 and IFN-α induced by Nagasaki infection, shown in Figure 9, were overcome by pre-treatment with Poly:IC, levelling up IFN-α in Nagasaki infected cells (*p* = 0.09) and IL-12 in cells infected with any *H. parasuis* strains (*p* = 0.10) (Figure 10).

## Discussion

In the present study, the immune response generated by porcine DC against *H. parasuis* non-virulent serovar 3 (SW114) or virulent serovar 5 (Nagasaki) alone or after a previous infection with H3N2 SwIV was evaluated. The goals in this study were the following: (i) to establish an in vitro model to study the interaction of virulent and non-virulent *H. parasuis* strains with DC that could give us some insight into the virulence associated with the immune response; (ii) to analyse whether SwIV infection could alter those responses.

Dendritic cells are competent antigen-presenting cells responsible for activation of naive T cells and generation of primary T-cell responses [31]. DC constitute the bridge between the innate and adaptive immune response [17]. The sentinel functions and the availability of DC beneath the epithelium of respiratory organs make them a suitable target for respiratory pathogens, such as SwIV or *H. parasuis* in pigs. Extensive studies on DC have been done in mice and humans and although the knowledge on swine immunology has been developing quickly in recent years, studies to highlight the interaction of pathogens with the porcine immune system are scarce. A recent study evaluated the ability of *Streptococcus suis* to interact with swine DC. Thus, *S*. *suis* capsular polysaccharide (CPS) was shown to interfere with DC phagocytosis and to be mainly responsible for DC activation, addressing the role of *S*. *suis* CPS as a critical virulence factor [32]. In a previous study, we have shown that phagocytosis resistance was a virulence mechanism of *H. parasuis*, on porcine alveolar macrophages (PAM). Although the Nagasaki strain entered the cells, it showed negligible association with PAM [8].

In the present study, we describe marked differences in the interaction of a virulent and a non-virulent strain of *H. parasuis* with porcine DC. Firstly, SW114 was rapidly internalised by poBMDC compared to Nagasaki and this ability was not affected by a previous infection with a H3N2 SwIV. The lower level of interaction of Nagasaki with poBMDC, as it was also observed in alveolar macrophages [8], could be a general evasion strategy from the immune system. On the contrary to *H*. *ducreyi* which persists in DC without affecting the eukaryotic cell viability [33], Nagasaki was killed once internalized in poBMDC. Although Nagasaki was internalised in lower numbers, it induced more cellular lesions when compared to SW114, indicating that virulence factors could be acting at this level. Although cell death after *H. parasuis* infection evaluated by the number of apoptotic and necrotic cells did not change regardless of single or co-infected experiments (data not shown), it seems that one strategy of the Nagasaki strain in interacting with swine DC may be to alter cell morphology to a certain extent without inducing increased cell death.

Antigen presentation, activation of co-stimulatory markers and signalling through released cytokines are three key points in triggering an effective immune response by APC. The reduction in SLA-II observed in H3N2 SwIV plus SW114 or Nagasaki-infected cells might be due to a reduction of an accessible surface membrane since SW114 was attached in large numbers at 1 and 8 hpi. However, after 8 h when the interaction of both bacterial strains with DC was similar, SLA-I expression decreased in H3N2 SwIV plus SW114-infected cells while increased in H3N2 SwIV plus Nagasaki infected cells. These differences in interaction of both pathogens with poBMDC might suggest that they interfere in a different way with the transport and/or recirculation of MHC I and II molecules. For example, it has been reported that *Helicobacter pylori* infect murine DC and as a consequence, MHC-II is retained within the *H. pylori* vacuoles and the export of MHC-II molecules to the cell surface is blocked [34].

The activation of APC is conditioned by the local environment in which they are primed, and this influences the way in which they control T helper type 1/type 2 (Th1/Th2) [35] or Th17 [36] cell development. In addition, adaptive immune response to micro-organisms is often characterised by the polarisation of the cytokine response. Generally speaking, type I cytokines are known to suppress type II responses and vice versa. Pathogens that strongly polarise the immune response may modify the type I and type II cytokine balance and/or effectors and consequently the local environment in which immunity to a concurrent micro-organism develops. In our system, swine DC infected with either of the *H. parasuis* strains tested showed a predominant inflammatory pattern of cytokines with high levels of IL-1β, IL-8, IL-6, IL-18 and TNF-α. Previously, Bouchet el al., reported that *H. parasuis* serotype 4 of moderate virulence, induced IL-8 and IL-6 secretion in higher levels compared to serotype 5 (high virulence) in newborn pig tracheal cells [37] while the same authors also reported that field strains of *H. parasuis* serotypes 4 and 5 induced similar levels of IL-8 and IL-6 [38]. The data presented here indicate that there is no difference in the capacity of SW114 (serovar 3, non-virulent) or Nagasaki (serovar 5, virulent) to induce IL-1β, IL-8, IL-6, IL-18, IL-10, IFN-α and TNF-α in porcine DC. Different cell types used in these studies may account for the above-mentioned apparent discrepancies.

There were significant differences regarding secretion of IL-12, the non-virulent strain being a higher inducer. IL-12 is a cytokine which links both innate and adaptive immunity systems playing a critical role in inducing Th1 responses, which in turn leads to the production of a number of cytotoxic cytokines, as well as interferon-gamma (IFN-*γ*) by T cells [39]. Therefore, differential secretion of IL-12 might be considered a candidate of virulence in terms of immune responses to *H. parasuis*. Further studies will elucidate whether this will be the case in pigs. Interestingly, when SwIV co-infection took place with Nagasaki, IL-12 secretion increased to levels comparable to cells infected with non-virulent SW114, suggesting that a change in IL-12 pattern was accomplished. IFN-α secretion may be due to viral PAMP through TLR3 or through bacterial PAMP due to TLR4. Nagasaki and SW114 strains differ in the nature of their lipooligosacharide (LOS) [40] and capsule production [8], nevertheless, IFN-α secretion was similar when a single bacterial infection took place. However, these structural differences may account for the different cytokine pattern in the co-infection experiment as well as acting as a positive feedback loop for interferon upon influenza infection. In mice, Nakamura et al. reported that co-infection with influenza virus and *Streptococcus pneumoniae* leads to synergistic stimulation of type I IFN [41], which might also be the case in the experiments presented in this work.

Recently also in mice, Negishi et al. demonstrated a modulated antibacterial T cell response as a result of cross-interference of RLR and TLR signalling pathways [42]. The authors demonstrated that TLR activation through IRF5 (bacterial infection) induced high levels of IL-12 and low levels of IFN-I whereas RLR activation through IRF3 (viral infection) induced high levels of IFN-I and low levels of IL-12. These pathways of activation could also apply in the data presented here, as SW114 was a high IL-12 and low IFN-α inducer and SwIV a low IL-12 and high IFN-α inducer in infected DC, as shown in Figure 9. Negishi et al. also reported that in the case of bacterial-virus co-infection, IRF3 is able to suppress IL-12. However, this was not the case in the experiments presented here, where in the co-infection or Poly:IC stimulation, IL-12 was not only inhibited but induced by the virulent Nagasaki strain. Differences in animal species and the degree of pathogen virulence used in these studies may account for the above-mentioned apparent discrepancies.

Overall, the results presented in this work show that in vitro DC studies may help to understand the complex relationship of virulent and non-virulent bacteria and the intimate relation among different pathogens in co-infections. These in vitro analyses allowed us to investigate new avenues for the stimulation of the immune system for better response to pathogens. In conclusion, we report for the first time immunological differences among virulent versus non-virulent *H. parasuis* strains in their interaction with DC and their modulation by SwIV co-infection.

## Competing interests

Sources of financial support have been acknowledged and the authors declare that they have no competing interest.

## Authors’ contributions

MM, VA, and TM designed the study, analysed and interpreted data. TM performed research and wrote the manuscript. CRC and ASC performed and analysed TEM data. JD critically reviewed the manuscript and contributed important intellectual output. LF performed the statistical analysis. MB sequenced the H3N2 SwIV. MCH performed intracellular survival assay. All authors contributed to editing of the manuscript and all authors read and approved the final manuscript.

## Supplementary Material

Additional file 1: Figure S1 H3N2 SwIV and H. parasuis SW114 or Nagasaki co-infected-poBMDC at (a) 1h and (b)8 hpi. Porcine BMDC were infected and stained using anti-SW14 or anti-Nagasaki rabbit serum 1 h at 4°C, and then with the anti-rabbit IgG-FITC antibody. Mock (grey histograms), SW114 (dotted line), Nagasaki (continuous line). Representative results of two independent experiments using poBMDC were derived from two animals. Click here for file

Additional file 2: Figure S2 Fluorescence images of co-infected-poBMDCs with H3N2 SwIV and SW114 or Nagasaki at 8 hpi. Porcine BMDC were infected and stained using (a) anti-SW14 or (b) anti-Nagasaki rabbit serum, and then with the anti-rabbit IgG-Dye Light 549, and anti-mouse NP HB65 ATCC antibody followed by an anti-mouse IgG-FITC. Therefore, nuclei were stained with DAPI (blue). SW114 or Nagasaki (red) and SwIV nucleoprotein (NP: green) Bar = 25 μm. Representative results of two independent experiments using poBMDC were derived from two animals. Click here for file

Additional file 3: Figure S3 TEM of poBMDC co-infected with H3N2 SwIV and SW114 at 8 h. Porcine BMDC showed many vesicles (ves) containing SW114 (a,b,c). Some vesicles had more than one bacteria (d, e). SW114 was found inside vesicles at different levels of degradation (asterisk, e, f, g). Also virus-like-particles (VLP) were observed (h, i). Bars: a = 2 μm; b-g = 200 nm; h = 100 nm and i = 50 nm. Click here for file

Additional file 4: Figure S4 TEM of poBMDC co-infected with H3N2 SwIV and Nagasaki at 8 h. Porcine BMDC were infected with SwIV followed by Nagasaki. At 8 hpi, poBMDC showed several vesicles (ves) (a-e). Few cells showed drastic cell damage (b). Some vesicles contained more than one bacterium (d, g). The Golgi of Nagasaki-infected poBMDC was dilated (f, h). Different levels of Nagasaki degradation were observed (black arrows) (h). The Golgi of Nagasaki-infected poBMDC was enlarged (i). Bars: a-c, g = 2 μm; d, f, i = 500 nm, e = 1 μm and h = 200 nm. Click here for file
