# Supplementary figures and images for: Differential interactions of virulent and non-virulent H. parasuis strains with naïve or swine influenza virus pre-infected dendritic cells
Source: Vet Res. 2012 Nov 16;43(1):80. doi: 10.1186/1297-9716-43-80 (PMC3585918; doi:10.1186/1297-9716-43-80)

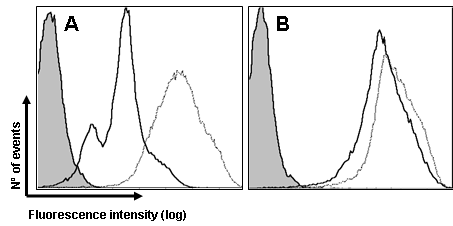

Supplement: Additional file 1: Figure S1 — H3N2 SwIV and H. parasuis SW114 or Nagasaki co-infected-poBMDC at (a) 1h and (b)8 hpi. Porcine BMDC were infected and stained using anti-SW14 or anti-Nagasaki rabbit serum 1 h at 4°C, and then with the anti-rabbit IgG-FITC antibody. Mock (grey histograms), SW114 (dotted line), Nagasaki (continuous line). Representative results of two independent experiments using poBMDC were derived from two animals. [file 1297-9716-43-80-S1.png]

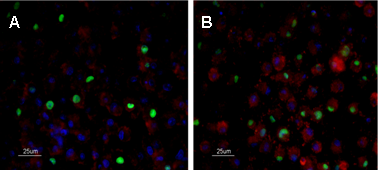

Supplement: Additional file 2: Figure S2 — Fluorescence images of co-infected-poBMDCs with H3N2 SwIV and SW114 or Nagasaki at 8 hpi. Porcine BMDC were infected and stained using (a) anti-SW14 or (b) anti-Nagasaki rabbit serum, and then with the anti-rabbit IgG-Dye Light 549, and anti-mouse NP HB65 ATCC antibody followed by an anti-mouse IgG-FITC. Therefore, nuclei were stained with DAPI (blue). SW114 or Nagasaki (red) and SwIV nucleoprotein (NP: green) Bar = 25 μm. Representative results of two independent experiments using poBMDC were derived from two animals. [file 1297-9716-43-80-S2.png]

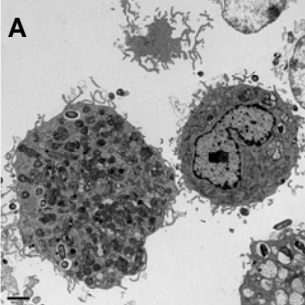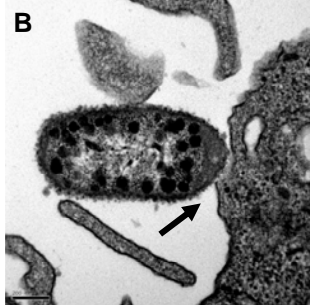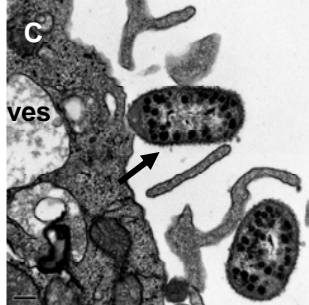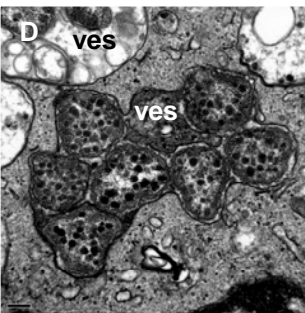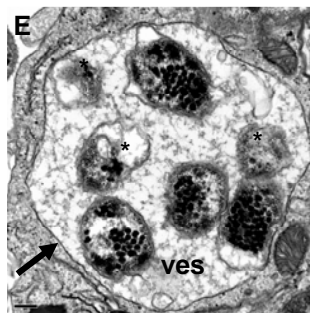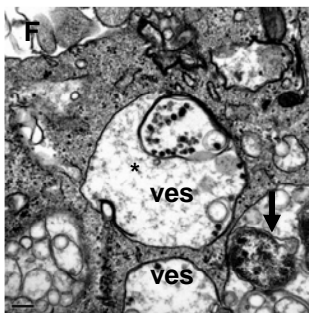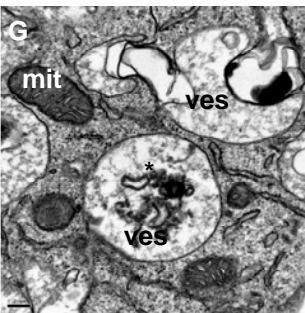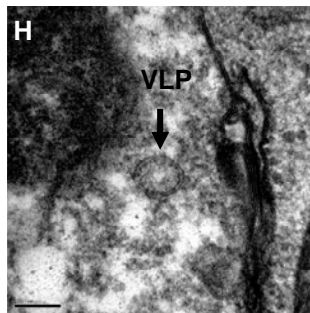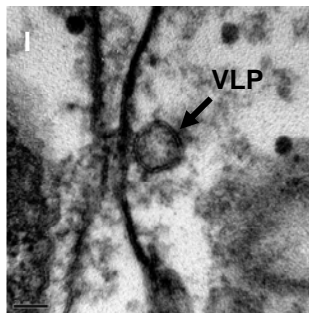

Supplement: Additional file 3: Figure S3 — TEM of poBMDC co-infected with H3N2 SwIV and SW114 at 8 h. Porcine BMDC showed many vesicles (ves) containing SW114 (a,b,c). Some vesicles had more than one bacteria (d, e). SW114 was found inside vesicles at different levels of degradation (asterisk, e, f, g). Also virus-like-particles (VLP) were observed (h, i). Bars: a = 2 μm; b-g = 200 nm; h = 100 nm and i = 50 nm. [file 1297-9716-43-80-S3.pdf]

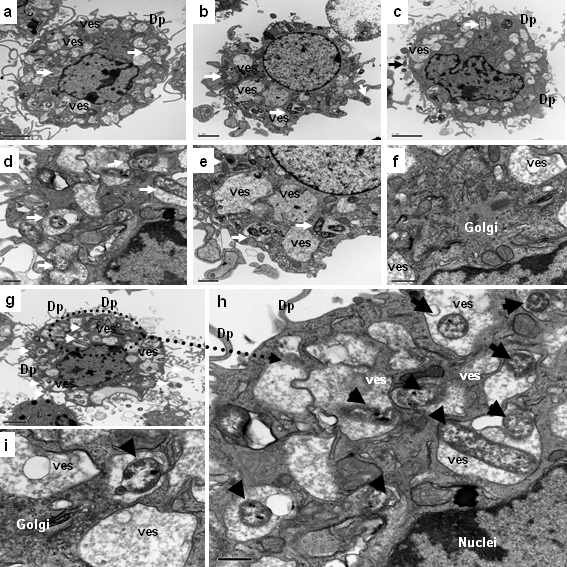

Supplement: Additional file 4: Figure S4 — TEM of poBMDC co-infected with H3N2 SwIV and Nagasaki at 8 h. Porcine BMDC were infected with SwIV followed by Nagasaki. At 8 hpi, poBMDC showed several vesicles (ves) (a-e). Few cells showed drastic cell damage (b). Some vesicles contained more than one bacterium (d, g). The Golgi of Nagasaki-infected poBMDC was dilated (f, h). Different levels of Nagasaki degradation were observed (black arrows) (h). The Golgi of Nagasaki-infected poBMDC was enlarged (i). Bars: a-c, g = 2 μm; d, f, i = 500 nm, e = 1 μm and h = 200 nm. [file 1297-9716-43-80-S4.png]
